# α-Terpineol: An Aggregation Pheromone in *Optatus palmaris* (Coleoptera: Curculionidae) (Pascoe, 1889) Enhanced by Its Host-Plant Volatiles

**DOI:** 10.3390/molecules26102861

**Published:** 2021-05-12

**Authors:** José Manuel Pineda-Ríos, Juan Cibrián-Tovar, Luis Martín Hernández-Fuentes, Rosa María López-Romero, Lauro Soto-Rojas, Jesús Romero-Nápoles, Celina Llanderal-Cázares, Luis F. Salomé-Abarca

**Affiliations:** 1 Postgrado en Fitosanidad, Programa de Entomología y Acarología, Colegio de Postgraduados Campus Montecillo, Km 36.5 Carretera, Texcoco 56230, Mexico; pinedarmanuel@gmail.com (J.M.P.-R.); rojo@colpos.mx (L.S.-R.); jnapoles@colpos.mx (J.R.-N.); llcelina@colpos.mx (C.L.-C.); 2 Instituto Nacional de Investigaciones Forestales, Agrícolas y Pecuarias, Progreso Número 5, Barrio de Santa Catarina, Delegación Coyoacán, Ciudad de México 04010, Mexico; hernandez.luismartin@inifap.gob.mx; 3 Postgrado en Edafología, Colegio de Postgraduados Campus Montecillo, Km 36.5 Carretera, Texcoco 56230, Mexico; rosal@colpos.mx

**Keywords:** terpenoid, signaling, chemical cue, kairomone, potentiation

## Abstract

The Annonaceae fruits weevil (*Optatus palmaris*) causes high losses to the soursop production in Mexico. Damage occurs when larvae and adults feed on the fruits; however, there is limited research about control strategies against this pest. However, pheromones provide a high potential management scheme for this curculio. Thus, this research characterized the behavior and volatile production of *O. palmaris* in response to their feeding habits. Olfactometry assays established preference by weevils to volatiles produced by feeding males and soursop. The behavior observed suggests the presence of an aggregation pheromone and a kairomone. Subsequently, insect volatiles sampled by solid-phase microextraction and dynamic headspace detected a unique compound on feeding males increased especially when feeding. Feeding-starvation experiments showed an averaged fifteen-fold increase in the concentration of a monoterpenoid on males feeding on soursop, and a decrease of the release of this compound males stop feeding. GC-MS analysis of volatiles identified this compound as α-terpineol. Further olfactometry assays using α-terpineol and soursop, demonstrated that this combination is double attractive to Annonaceae weevils than only soursop volatiles. The results showed a complementation effect between α-terpineol and soursop volatiles. Thus, α-terpineol is the aggregation pheromone of *O. palmaris*, and its concentration is enhanced by host-plant volatiles.

## 1. Introduction

Most of the members of the Curculionidae family, except for Platypodinae and Scolytinae, are called weevils. This term comes from their characteristic long snouts and capitate antennae with small clubs [1]. When not in use, the antennae are stored in grooved cavities along the snout. As mentioned, these insects possess a long rostrum (snout) with mouthparts at the end of it. [1,2]. Their distinctive snout allows feeding on various plant organs like roots, stems, leaves, flowers, and fruits [1]. Because of the damage caused to diverse plant species, several weevils are considered economically important agronomic pests [1,3,4,5]. In addition to damage from their feeding behavior, female weevils could oviposit in holes left after feasting on the fruits; thus, new-offspring will feed on the fruit mesocarp and seeds, causing their detachment [6].

In Mexico, several curculios such as *Scyphophorus acupunctatus*, *Rhynchophorus palmarum*, *Anthonomus eugenii*, and *Anthonomus grandis* are considered principal crop pests, and they have been extensively studied; these insects attack agave, coconut palm, chili pepper, and cotton plants, respectively [7,8,9,10]. Weevil pests also attack soursop (*Annona muricata* L.), another important Mexican agronomic product. Mexico is the biggest soursop producer worldwide [11,12]; however, the Annonaceae fruits weevil (*Optatus palmaris* Pascoe) causes significant losses in this crop. This weevil feeds on young leaf buds and flowers, causing detachment from the tree [6]. Annonaceae fruits weevils also feed on fruits, preferring ripe fruits if available, causing external damage to up to ca. 40% of the fruit surface. Additionally, larvae instar feed from the inside of the fruit, destroying the mesocarp and the seeds of the soursop [6]. The weevil attack can also cause detachment of small fruits from the tree [6,13,14]. 

The life cycle of the Annonaceae fruits weevil last around 215 days; five days as egg, 73 days as larva, 25 days as pupa, and 112 days as an adult. In Mexico, their activity period is reported to be between 8:30 h and 23:00 h for adult specimens [15]. However, another report mentioned that the activity period of this weevil is restricted between 10:00 h and 18:00 h in the field [16]. The presence of adults in the field starts with the beginning of the rainy season (August–November). Once in the field, the adults feed and copulate on ripening soursop fruits, especially close to their harvest point. It is common to find up to 30 adults per fruit or even more depending in the size of the fruit. On the other hand, when they are present in leaf buds and flowers, usually only one specimen is found in those organs. When not copulating, the females mainly dedicate to feed and oviposit. Conversely, the males go to the treetop to rest and feed on young leaf buds and flowers before copulating and feeding on soursop fruits again [15]. Other Annonaceae fruits weevils’ alternative host-plants are reported to be cherimoya (*Annona cherimola* Mill), ilama (*Annona macroprophyllata* Donn. Sm.), and custard apple (*Annona reticulata* L.) [17].

Due to its recent detection and fast spread, research on the Annonaceae fruits weevil control is limited to some insecticides rotating use [18]. However, this strategy is not enough to fully control the weevil population level and, thus, the damage caused by them. Typically, effective monitoring and control of weevils include the use of insect traps baited with their aggregation pheromones [19]. The use of semiochemicals could potentially handle or even fully control Annonaceae fruits weevil infestations. However, research about pheromones or kairomone does not exist for this insect species.

Therefore, the feeding behavior of males and females of the anonacea weevil was characterized. The characterization consisted of behavioral assays carried out by olfactometry. Subsequently, chemical analyzes were carried out to identify candidate volatiles functioning as chemical cues. We expected results to provide insight on volatiles mediation of the feeding behavior of the Annonaceae fruits weevil adults. Moreover, the obtained data may serve as the basis for establishing volatile-based management of this pest.

## 2. Results

**Figure 1:**
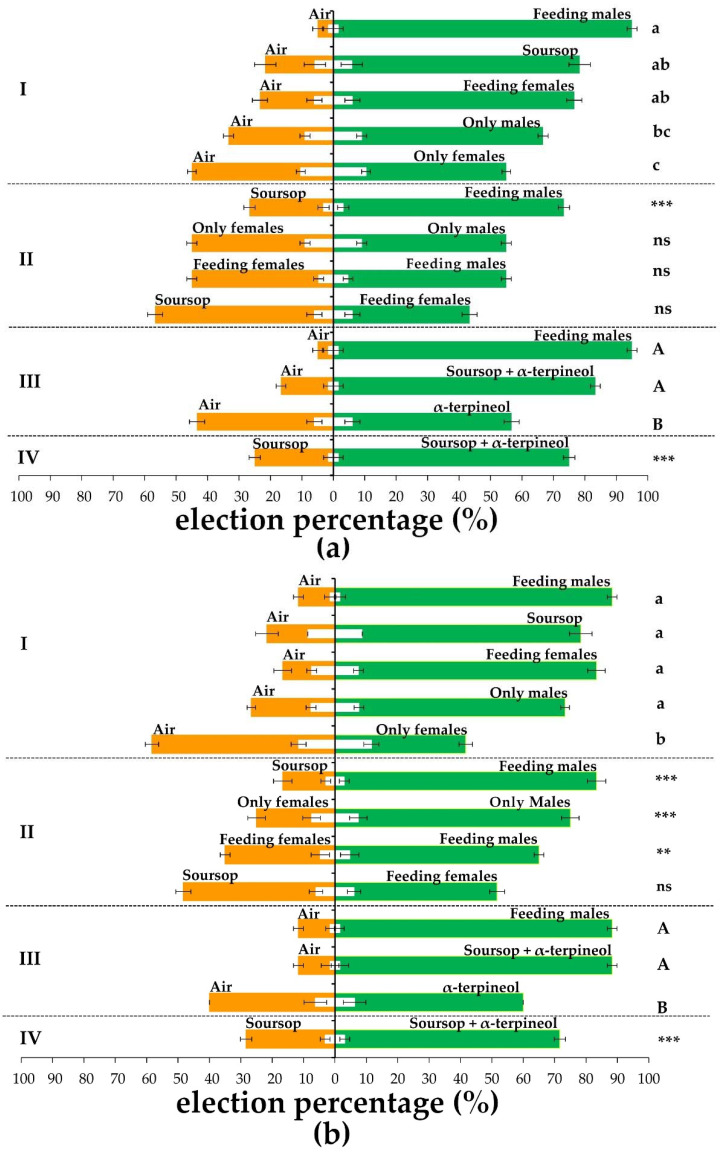
Olfactory preference of males and females of *Optatus palmaris* to different odors as single or double stimulus sources. (**a-I**), male preferences to single stimulus source vs. air. (**a-II**), male preference when two odor source are challenged against each other. (**a-III**), male preference to one stimulus source involving α-terpineol against air. (**a-IV**), male preference to soursop challenged against soursop supplemented with α-terpineol. Data represent the selection percentage of males to an odor stimulus (*n* = 4). Horizontal black bars indicate standard error values. Treatments with the same letters are not significantly different in a (**a-I**) Tukey test (*p* < 0.05), (**a-II**) G-test (G < 0.05), *** < 0.0001, (**a-III**) Tukey test (*p* < 0.05), and (**a-IV**) G-test (G < 0.05), *** < 0.0001. (**b-I**), female preferences to single stimulus source vs. air. (**b-II**), female preference when two odor source are challenged against each other. (**b-III**), female preference to one stimulus source involving α-terpineol against air. (**b-IV**), female preference to soursop challenged against soursop supplemented with α-terpineol. Data represent the selection percentage of males to an odor stimulus (*n* = 4). Horizontal black bars indicate standard error values. Treatments with the same letters are not significantly different in a (**b-I**) Kruskal–Wallis/Bonferroni test (*p* < 0.05), (**b-II**) G-test (G < 0.05), ** < 0.001, *** < 0.0001, (**b-III**) Kruskal-Wallis/Bonferroni test (*p* < 0.05), and (**b-IV**) G-test (G < 0.05), *** < 0.0001. The statistical analysis was performed separately for males’ and females’ data. White bars represent average values (*n* = 4) of non-responding insects registered in each preference test. Horizontal black bars indicate standard error values. The non-responding insect values were compared for the first three sections (I, II, and III) with an ANOVA test. There were no statistical differences among non-responding insect values in any set of experiments analyzed (*p* > 0.05).

The Annonaceae fruits weevil has a long-life cycle, and an artificial diet for its rearing is lacking. Therefore, this research employed wild insects. On the first experimental stage, olfactory two-choice preference experiments were carried out in males and females. Before starting any odor testing, the air in both olfactometer arms was used to check no specific selection by male or female weevils existed. In general, only 6–13% (1 to 2 weevils out of 15) chose an olfactometer arm; the rest showed no response to air stimulus. Similar results were observed for the carrier solvent (hexane) evaporation tests. These selection trials indicated no position, light, or remnant volatile or solvent effects over the behavior of the tested weevils. After the blank test, different odor sources vs. air were used as preference tests separately in male and female weevils. Treatments included pieces of soursop, only non-feeding males, only non-feeding females, males feeding on soursop (feeding males), and females feeding on soursop (feeding females). Results showed that feeding males produced the most attractive aroma for male weevils with 94.99% selection (F_4,15_ = 11.77, *p* < 0.001). On the other hand, the preference of males towards soursop and feeding females showed non-significant statistical differences (F_4,15_ = 11.77, *p* < 0.001) among treatments (Figure 1). Conversely, females’ preference towards feeding males, soursop, and feeding-female volatiles showed non-significant statistical differences among them. That is, female weevils did not display a specific behavior for each tested volatile source. Finally, both males and females showed less preference by volatile emitted by non-feeding females (F_4,15_ = 11.77, *p* < 0.001; F_4,15_ = 26.44, *p* < 0.0001, respectively). 

Based on this information, in a second experimental stage, both males’ and females’ preference to soursop volatiles was challenged against volatiles from feeding males and females. Moreover, both sexes’ preference to volatiles simultaneously emitted by feeding males and feeding females from two different emission arms was tested. Finally, the preference for non-feeding males and non-feeding females was evaluated too. The preference of aroma of feeding males was highly significant (G = 22.64, *p* < 0.0001 by males and G = 48.50, *p* < 0.0001 by females) by both sexes (Figure 1). Females displayed marked preference to the treatments containing male volatiles, especially to those of feeding males. Even so, males did not display significantly differentiated preference when both feeding females and feeding males are simultaneously tested (G = 0.99, *p* = 0.31) (Figure 1). Thus, the higher preference for feeding males and soursop aromas observed in the first and second olfactometry assays demonstrated the presence of an aggregation pheromone and a kairomone which could parallel mediate the feeding behavior of the Annonaceae fruits weevil. All of the non-responding insect values in each experimental set did not show significant differences when analyzed by an ANOVA test. This might mean that the no response behavior of some weevils are related to some intrinsic biological factors of each insect rather than the strength of the response evoked by every odor source (Figure 1). 

**Figure 2:**
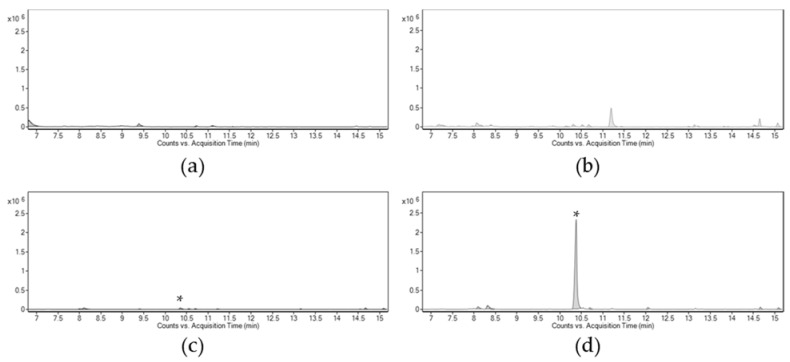
Gas chromatography-mass spectrometry profiles of volatile blends produced by different odor sources. (**a**), soursop, (**b**), insect females feeding on soursop, (**c**), non-feeding males, and (**d**), males feeding on soursop. * indicates the presence of an exclusive peak at 10. 34 min found in males and increased when males feed on soursop.

Based on these results, volatiles emitted by soursop, only males, only females, and feeding females were used as background chromatograms to scrutinize volatiles specifically produced when males feed on soursop, which could potentially function as aggregation pheromone. The volatile fractions were trapped by solid phase microextraction (SPME) and dynamic headspace (DH). Comparing the GC-MS profiles of all of the samples showed the presence of a chromatographic peak exclusively found in males at 10.34 min (Figure 2). Remarkably, this peak area increased from five to fifty times in some individual cases and resulted in a 15-fold average increment of the content of this compound when males fed on soursop (Figure 2). The chromatographic peak was identified as α-terpineol [2-(4-methylcyclohex-3-en-1-yl)propan-2-ol] by matching its spectrum (95%) with that of the NIST library.

**Figure 3:**
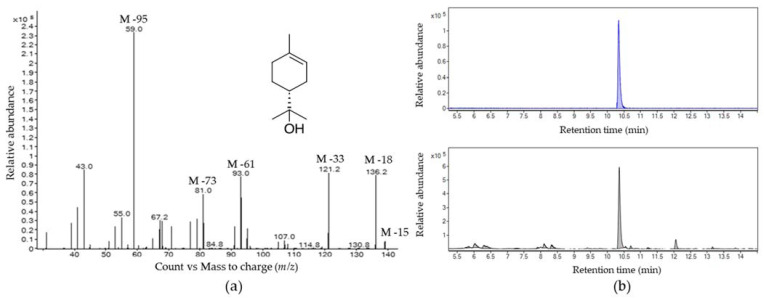
Gas-chromatography-mass spectrometry identification of α-terpineol (**a**), mass spectrum obtained from a chromatographic peak of the volatile blend produced by males feeding on soursop. The matching factor with the library spectrum (NIST 2014) of this metabolite was 95%. (**b**), Retention time comparison between chromatographic peaks of a standard compound (upper chromatogram) and a volatile blend produced by males feeding on soursop (lower chromatogram).

Compound identification was also supported by the interpretation of mass losses when looking at the mass spectrum of such metabolite. The main *m*/*z* values were 139, 136, 121, 93, 81, 67, and 59. Based on these values, a total loss of the parent and molecular ions was determined. Furthermore, the *m*/*z* 139 corresponded to M-15, which is explained by the loss of a methyl group from the molecule. The *m*/*z* 136 corresponded to M-18 explained by a water molecule loss typical in alcohols, in this case, α-terpineol. This fact was also supported by *m*/*z* 121 (M-33), which indicated the loss of a methyl group and a water molecule typical in alcohols with a methyl group in its chain aliphatic side. The *m*/*z* 93 indicated the formation of a C_7_H_9_^+^ ion which is a typical mass for terpenoids [20,21]. The *m*/*z* 81 was explained by the loss of C_4_OH_10_^+^, potentially followed by a Mclafferty rearrangement. The *m*/*z* 69 value was also explained by the loss of all radicals bounded to the cyclohexene ring of α-terpineol, followed by its rupture through a retro Diels–Alder reaction. The base peak corresponded to *m*/*z* 59, representing a C_3_H_7_O^+^ fragment, which is also a typical mass for alcohols [21] (Figure 3a). Finally, the chromatographic peak’s retention time and mass spectrum were compared with those of a standard compound (Figure 3b).

To further correlate the release of α-terpineol with the feeding behavior of the Annonaceae fruits male weevils, its concentration in the headspace was quantified. Thus, matrix recovering effects were determined in order to achieve proper quantitative data. Briefly, some paper disks were loaded with a known concentration of the volatile, then trapped, extracted and quantified. Moreover, the same process was performed with Tenax^®^ cartridges directly loaded with the same compound. The average α-terpineol content of the filter paper extracts was 14.89 ± 2.61 ng/µL, and 14.29 ± 3.18 ng/µL for the cartridges directly loaded with the same amount of α-terpineol. There were no significant differences between these values (*t* > 0.05). Thus, the matrix recovering effect experiments showed that all of the α-terpineol originally loaded in the disks filter papers (18 µg) released to the headspace and moved into the cartridge. However, the concentration of the filter papers extracts (14.89 ± 2.61 ng/µL) and its total extract volume (350 µL), indicate that only 5.21 µg of α-terpineol were recovered from the cartridge under the used elution conditions. That is, 71% of the total amount of this metabolite is kept in the dynamic headspace cartridge. Thus, every initial quantification value was compensated with 71% of its mass. 

**Figure 4:**
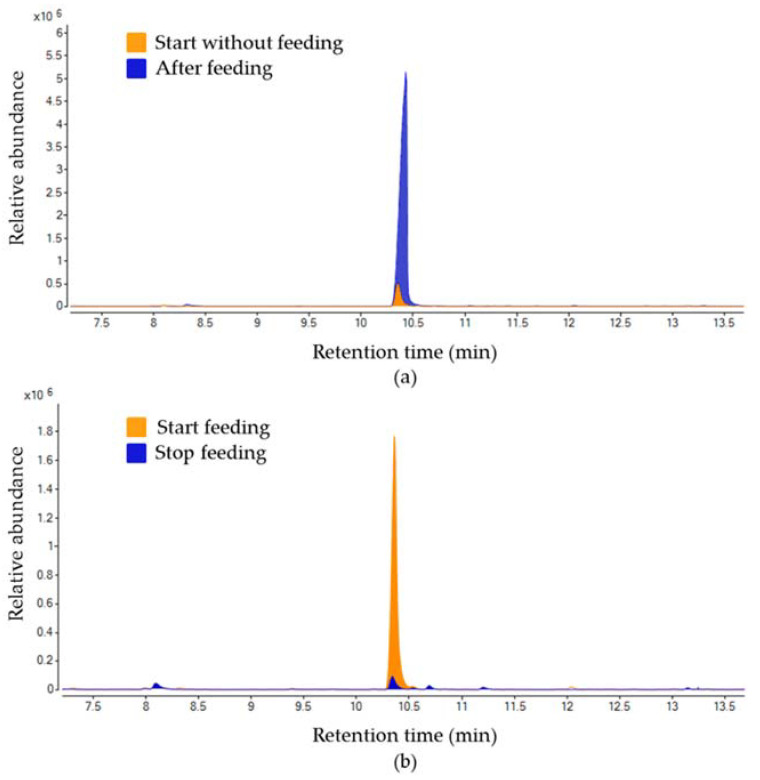
Representative chromatograms of the feeding and starvation effects over the α-terpineol release by *Optatus palmaris*. (**a**), Increase of the production of α-terpineol after *O. palmaris* males feed on soursop. (**b**), Decrease of the production of α-terpineol after *O. palmaris* males stopped feeding on soursop.

Subsequently, a set of reversal feeding experiments (feeding-starvation) were performed. Briefly, the production of α-terpineol was determined in males initially feeding on soursop. After 48 h, the food was removed and the α-terpineol production was quantified again. The same experiment was simultaneously performed with males without food and fed with soursop after 48 h. These experiments allowed to characterize the release of α-terpineol in response to the feeding habits of the Annonaceae fruits male weevils. Interestingly, as previously observed, the release of α-terpineol was higher in the initial feeding-males. On the other hand, the non-feeding insects increased this volatile production when fed and, conversely, the initial feeding males reduced their α-terpineol release when food was removed. Regardless of the total amount of this compound produced by non-feeding males fed after a starvation period, the release of this volatile increased 87 ± 3.87% in all the experiments (Figure 4). The same decrease ratio was observed when food was removed from initial feeding males (Figure 4). 

Considering the total amount of males (40) used in the bioassays to get the averaged α-terpineol content in the headspace, a release rate of 1.83 ± 0.36 ng per 40 males per second was determined. When considering the production per one equivalent male, a release ratio of 0.05 ± 0.01 ng per equivalent male per second was determined. Thus, solutions ranging between 0.5 and 10 ng/µL and 100 ng/µL were tested in two-choice olfactometry experiments. The range 0.5–2 ng/µL showed no clear selection pattern neither by male nor female weevils. The range 3–5 ng/µL showed attraction to both male and female weevils. Above these concentrations, a repellent effect took place for both sexes, especially at 100 ng/µL. Because of this, all following preferences experiments with this metabolite used solutions at 4 ng/µL. Intriguingly this volatile compound showed only 56.66% and 60% of attraction to males and females, respectively. Therefore, even if all evidence showed this volatile as a metabolite related to the feeding behavior of the Annonaceae fruits weevil, its not-very-high attractiveness indicates rather the presence of other possible volatile compounds potentiating its attraction effects. This volatile compound(s) could be produced either by the insect or by its host-plant. However, the chromatographic scrutiny of the analyzed samples did not show any other potential compound working as a secondary component of the aggregation pheromone of the Annonaceae fruits weevil. Thus, the whole volatile bouquet of soursop was supplemented with 4 ng/µL of α-terpineol, which simulated the effect of feeding males on this fruit. The combination of the soursop volatiles and this metabolite resulted in the increased attraction of males (83.33%) and females (88.33%) to this odor source. Moreover, these values are comparable to those obtained from feeding males showing non-significant statistical differences between them (*p* > 0.05) (Figure 1). Furthermore, when the aroma of soursop and soursop supplemented with α-terpineol were challenged against each other, the latter was at least two times more attractive to both male (74.99%) and female weevils (71.66%) (Figure 1).

The GC-MS analysis of the volatiles fraction trapped by dynamic headspace and solid phase microextraction from soursop fruits, detected more than 200 chromatographic peaks. Unfortunately, just few of them showed a roughly good identification match with the NIST library (>75%). A total of 16 compounds were identified including mono- and sesquiterpenoids, aldehydes, fatty acids, a ketone, and an alcohol. Nonetheless, only three compounds, D-limonene, *β*-caryophyllene, and nonanal were annotated by comparison with standard compounds (Supplementary Table S1).

## 3. Discussion

In nature, chemical messengers constitute the language of intra- and inter-kingdom communication [22]. Several of these chemical cues possess a volatile character, producing physiological or behavioral responses in the receptor organism [23]. Volatiles that determine intraspecific interactions are labeled pheromones. Depending on the behavioral outcome, they can be categorized as sexual, aggregation, alarm, attack, or epideictic pheromones, among others [24]. Currently, these volatile compounds, when used in combination with traps, efficiently and effectively detect, monitor, and control insect pests [25]. 

In an era where resistance to conventional control strategies and chemicals by microbes and insects increases continuously, alternatives become essential to ensure food safety. There are several successful cases of control in this context, especially in lepidopterans and curculios insects, with semiochemicals [9,26]. In this research, volatile compounds mediating the feeding behavior of the Annonaceae fruits weevil (*Optatus palmaris*) were scrutinized for their potential use in managing this insect pest. The first experimental stage showed high preference by male and female weevils to the aroma of soursop and males feeding on this fruit. Our research showed weevils use soursop volatiles to find food based on the attraction to soursop aroma; this behavior resembles behavior caused by kairomone odor [27]. However, host-plant volatile compounds are not the only cue used for food or mate location, as observed in some weevil species. For example, male-produced aggregation pheromones cause attraction and grouping of males and females of the same species to the food source for feeding and mating [19]. The same behavior was recorded in the olfactometry assays as feeding males stimulated higher aroma preference, particularly by male weevils, compared to any other odor source (Figure 1). Lower preference for feeding females further corroborated the results. The weevil attraction to this treatment could result from soursop volatiles only, as showed by the comparison of attraction values of feeding females and soursop tested against air (Figure 1). In the case of females, even if non-significant differences were observed between preferences by feeding males or only soursop volatiles, attraction to feeding males showed a trend to be more preferred. However, the observed values also indicated females possess high host volatiles usage to locate food. In this regard, it has been reported that female weevils locate their host-plant using various volatile cues, including kairomone and aggregation pheromones [28,29]. 

The observed insect behavior suggested the convenience of volatile chemical analysis. In several weevil species, aggregation pheromones are molecules containing ten carbons or less [7,8,9]. Because of this, solid-phase microextraction (SPME) polydimethylsiloxane (PDMS) and a carboxen/divinylbenzene in PDMS fibers were chosen to capture volatiles in the 40–275 g/mol range. Dynamic headspace (DH) experiments complemented the SPME data. The chromatographic analyses of all volatile fractions provided one distinctive chromatographic peak, highly amplified only in feeding males. That is, a low-concentration metabolite produced by groups of non-feeding males averaged a 15-fold increase when males fed on soursop. This metabolite was identified as α-terpineol, a common plant monoterpenoid.

It has been reported that some insects can hijack host-plant metabolites for direct or indirect defense or communication molecules [30]. However, GC-MS analyses did not detect α-terpineol (limit of detection = 2.10 ng/µL), in the volatile makeup of soursop. The production of this metabolite by the soursop fruits after damage caused by feeding of the weevils was discarded as the chromatographic profiles of the females feeding on soursop also did not show α-terpineol. Therefore, de novo synthesis by the Annonaceae fruits weevil must occur. Compared to the thousands of terpenoids detected in plants, these metabolites are found only in nine orders of insects [31]. Thus, the total amount of insect-produced terpenoids represent less than 1% of all terpenes found in nature [32]. Coleoptera to which the anonacea weevil belongs is one of these nine orders capable of synthesizing *de novo* terpenoids [31]. Specific examples of insects producing terpenoids as aggregation pheromones components in the Curculionidae family are *Anthonomus eugenii*, *Anthonomus musculus*, *Anthonomus rubi*, and *Anthonomus grandis* [33,34,35]; these insects attack chili pepper, cranberry, strawberry flowers, and cotton bolls, respectively. Their aggregation pheromones contain terpenoids such as geranic acid, geraniol, grandlure II, III, IV, and lavandulol [33,34,35]. Furthermore, *Curculio caryae* uses some of these compounds at varying ratios, as aggregation pheromone [36]. To our knowledge, α-terpineol is present only in the cerambycid beetle *Megacyllene caryae* as one of the seven components of its aggregation pheromone [37,38]. Hence, this is the first report α-terpineol is potentially produced as a unique aggregation pheromone component in the Curculionidae family. 

Additionally, a set of reverse feeding experiments showed an increased-decreased release relationship of α-terpineol with the feeding behavior of the Annonaceae fruits weevil. These results further supported this metabolite acts as an aggregation pheromone [19,39,40] (Figure 4). Moreover, during insect collections, observations in the field also confirmed that soursop serves as a copulation and feeding site for this weevil. This in-field behavior corresponds to the typical behavioral pattern of insects under an aggregation pheromone effect [19]. 

However, α-terpineol showed no strong attraction effect to males or females when individually tested against air. The attraction values of this volatile were around 60% for both sexes. Moreover, the tested solution acted as a repellent when the concentration increased above 10 ng/µL. The repellent effect was powerful at 100 ng/µL: all specimens, independently of the sex, selected the air arm of the Y-olfactometer. This behavior does not indicate a preference for air, but rather, it suggests an escape from the concentrated airstream with α-terpineol. The concentration-related anti-aggregation effect of α-terpineol may work as follows: when there is not enough space for feeding and mating because of high insect density in a fruit or tree, a high α-terpineol concentration from the cluster of weevil males signals other conspecifics the lack of space availability [19]. Anti-aggregation effects mitigate intraspecific competition for food and mating [41]. Thus, using a single compound, which role depends on its concentration, represents an advantageous, energy-saving strategy.

The observed, relatively low attraction effect of α-terpineol (60%) indicated that the aggregation effect signaled by feeding males depended on other volatile compounds too. As previously mentioned, GC-MS analyses discarded another compound’s possibility complementing the attraction effect of the aggregation pheromone. The combination of α-terpineol with soursop volatiles explored a second possibility. The blend attracted around 40% more than only α-terpineol when challenged against air. Moreover, in a dual choice assay, soursop volatiles supplemented with α-terpineol challenged against only soursop volatiles were twice as attractive to both male and female weevils. 

Previous research reported that host-plant volatiles enhance some insect pheromones’ activity [42]. In this regard, interaction of volatile compounds from host-plant and insects and its repercussion in the resulting attractiveness have been considerably studied in Coleoptera [31]. For example, boll weevil (*A. grandis*) captures in traps increased significantly when its aggregation pheromone was combined with *trans*-2-hexen-1-ol, *cis*-3-hexen-1-ol, or 1-hexanol; compounds emitted by cotton plants [43]. The same effect happened in traps baited with the plum curculio aggregation pheromone and benzaldehyde, a fruit volatile [44,45]. Similarly, host-plant volatiles synergized the response of the Asian palm weevil (*Rhynchophorus ferrugineus*), the South American palm weevil (*Rhynchophorus palmarum*), and the agave weevil (*Scyphophorus acupunctatus*) [46,47]. These effects are not limited to agronomic crop pests but also extended to forest insects. The captures of traps baited with the aggregation pheromone of *Dendroctonus ponderosae* increased highly (5- to 13-fold) when combined with pine volatiles [48]. 

Hence, we confirmed α-terpineol as a volatile compound linked to the feeding behavior of males of the Annonaceae fruits weevil. Furthermore, this volatile is used as an aggregation pheromone, which host-plant volatiles enhance its effect. Further field trials corroboration might be required, but these results highlight the potential of using semiochemicals associated with the feeding behavior of the Annonaceae fruits weevil for its management and control. 

However, even if the GC-MS analysis of the volatile fraction of soursop fruits detected more than 200 chromatographic peaks, just few of them showed a good identification match with the NIST library. Only three compounds, D-limonene, *β*-caryophyllene, and nonanal were annotated by comparison with standard compounds (Supplementary Table S1). Furthermore, there was no clear criteria of selection for specific soursop volatile selection for further combinatorial experiments. Moreover, even if identified, D-limonene, and *β*-caryophyllene are general compounds found in several plant species [49,50], which make them not really soursop specific compounds. These findings suggest the need for developing a specific approach for the characterization of the Annonaceae fruits weevil’s kairomone. This, specially to determine specific compounds and ratios which enhance the aggregation pheromone attractiveness. 

## 4. Materials and Methods

### 4.1. Insect Collection and Species Corroboration

The insects were collected at the municipality of Compostela, Nayarit, México (21°6′17.337″, −105°9′49.917″; 21°6′2.808″, −105°10′40.872″; 21°6′3.8874″, −105°9′48.492″; 21°6′13.572″, −105°9′52.2″). *Optatus palmaris* males and females were manually collected during August 2019 and September 2020. The insects were collected and transported on hermetic plastic bottles (1 L). The bottles were pierced to allow gas exchange for insects’ oxygenation. Subsequently, the specimens were sexed according to their morphological features [51]. The weevils were separated by sex and kept separated in entomological cages. They were fed with soursop pieces, and their rearing conditions were set to 25 ± 2 °C, relative humidity between 60 and 70%, and a photoperiod of 12:12 (L:D). The species corroboration was made using morphological characters previously reported [51]. The identified specimens were deposited at the Insect Collection of the Colegio de Postgraduados (CEAM). 

### 4.2. Volatile Capture

Volatile compounds were trapped by solid-phase microextraction (SPME) and dynamic headspace (DH). In both techniques, borosilicate flasks were employed. Material cleaning was performed by subsequent washes with 2% Extran^®^, distilled water, acetone, and left to dry in a fume hood. Afterward, the material was heated at 300 °C for 3 h. 

#### 4.2.1. Solid-Phase Microextraction 

Groups of 40 insects, only males or only females, were placed in 250 mL SPME flasks. Polydimethylsiloxane (PDMS/100 µm) and Divinylbenzene/Carboxen/PDMS (50/30 µm) fibers were used to trap volatiles. Both fibers were 1 cm long. Before volatile collection, the SPME fibers were cleaned in the injection port of a gas chromatograph (GC) (HP-6890, Midland, ON, Canada) at 250 °C. Subsequently, the SPME fiber was introduced through the flask septa, which contained a metallic mesh envelope to avoid contact between insects and the fiber. The distance between the SPME fiber and the insects was 2 cm. The volatile collection time was 1 h. Subsequently, the fiber was removed and desorbed in the GC injection port at 250 °C for 2 min for chromatographic analysis. The volatile samples were taken from groups of only males, only females, males feeding on 3 g of soursop, females feeding on 3 g of soursop, and only soursop. Blanks contained volatiles trapped from empty SPME flasks. Ten replicates were performed for each group. 

#### 4.2.2. Dynamic Headspace 

Groups of 40 insects, only males, only females, males feeding on 8 g of soursop, females feeding on 8 g of soursop, and only soursop were placed in 650 mL Drechsel gas washing bottles (PIREX^®^, glendla, AZ, USA, EE.UU). Airflow was provided by an air pump (ELITE 802^®^, Colchester, VT, USA) attached to volatile-free PVC tubing (Nalgene^®^, Rochester, NY, USA, 180 PVC, 3/16″ ID). The airstream was filtered with a 50 mg Tenax^®^ Baltimore, MD, USA, (60/80) cartridge. The airstream was humidified with a soft bubbling of distilled water. The airflow entering the system was 0.330 L/min controlled with a flowmeter (GILMONT^®^, London, UK) and calibrated with a manual glass flowmeter (Hewlett-Packard). The volatiles carried out by the airstream were finally captured in a cartridge containing 50 mg of Tenax^®^ (60/80) as adsorbent and 20 mg of glass wool at each end of the cartridge. All of the cartridges were previously washed with 5 mL of hexane and left to dry in a fume hood, to be then heated at 300 °C for 3 h. The volatile collections were carried out for 48 h at the same temperature, humidity, and light conditions as the rearing conditions. Once the volatile collection time was reached, cartridges were eluted with 350 µL of HPLC-grade hexane. Then, 50 µL of hexane were added to increase the recovered volume. Blanks consisted of the elution of cartridges connected to empty bottles. Ten replicates were performed for each group. 

### 4.3. Gas Chromatography Coupled to Mass Spectrometry (GC-MS) 

The volatile extracts were analyzed with an HP-6890 gas chromatograph coupled to an HP-5973 single quadrupole mass spectrometry (MS) detector. The GC-MS system was equipped with an HP-5MS column (30 m × 0.250 mm ID, and 0.25 μm stationary phase thickness, J&W Science, Folsom, CA, USA) for sample separation. Helium (99.999% purity) was used as carrier gas at a flow rate of 1 mL/min for all analyses. The oven was programmed to start at 60 °C for 1 min, then increased 8 °C/min up to 90 °C followed by a 1 min hold. Subsequently, the temperature increased again at 5 °C/min up to 190 °C and was held by 1 min. Finally, the temperature increased 10 °C/min up to 250 °C. The injection port was set to 250 °C on splitless mode. The injection volume was 1 μL. For liquid injection and SPME injection, 2- and 0.75-mm ID liners were used, respectively. The ion source and quadrupole temperature of the mass detector were 230 °C and 150 °C, respectively. The transfer line temperature was 280 °C. Ionization energy in EI mode was 70 eV, and the mass data was acquired on SCAN mode (50–550 *m*/*z*). Chromatographic peak identification was made by comparing their ion spectra with the NIST library (version 2014) and comparing retention time and spectrum with that of a standard compound.

A calibration curve was built to quantify the α-terpineol content in the samples. The concentrations ranged from 2.6 to 1984 ng/µg. Three replicates were made for each point of the calibration curve, and the average value of each was used to construct a final calibration curve for sample quantitation. Linear regression was calculated from the areas of the injected standard compound solutions; the slope equation was obtained, and the area of the samples was used to determine their concentration. The concentration was expressed on ng of α-terpineol per equivalent male per second. 

#### 4.3.1. Matrix Recovering Effects

To evaluate possible matrix effects, dynamic headspace experiments were set. First, filter papers (4 cm diameter) were loaded with 18 µg of α-terpineol. Subsequently, the paper disks were placed in 650 mL Drechsel gas washing bottles (PIREX^®^, EE.UU). The terpineol in the paper disks were trapped in the same manner as for previously described biological samples (*n* = 4). A second experiment was carried out by loading 600 µg of α-terpineol contained in 50 µL of hexane in a cartridge with 50 mg of Tenax^®^ (60/80) as adsorbent and 20 mg of glass wool at each end. The solvent was left to dry for 3 min and the compound was eluted as previously described for other biological samples (*n* = 4). Extracts from both experiments were quantified and compared against each other to check if all the α-terpineol was moved from the headspace to the Tenax^®^ cartridge. That is, all the α-terpineol was considered to be moved from the headspace to the cartridge if no significant concentration differences between both extracts were determined. To check matrix effects from the Tenax^®^ cartridge, the total amount of α-terpineol in the whole eluted extract was compared to that of the initial amount loaded in the paper disk of this metabolite (*n* = 4). The analysis showed that an average of 71% (*n* = 4) of the total amount of α-terpineol was retained in the cartridge under the elution conditions used in the experiments. This matrix effect was corrected during the calculation of the α-terpineol content by adding this percentage (71%) in the resulting concentration of the samples.

#### 4.3.2. Limit of Detection (LOD) and Limit of Quantification (LOQ)

To determine the LOD and LOQ values for α-terpineol, a 15 points calibration curve ranging from 0.04 to 627.52 ng/µg was constructed. However, only the first 12 points which showed linearity (0.04−79.04 ng/µg). The R^2^ coefficient for this linear regression was 0.994, and the slope equation was y = 72391x + 1077.9. The typical standard error xy (Sty) and slope (m) values were calculated for the resulting data set with the “SLOPE” and “STEYX” functions in Microsoft Excel^®^ 2016. The values were used in the following formulas: LOD = 3.3 (Sty/m) and LOQ = 10 (Sty/m).

For this study, the LOD value was equal to 2.10 ng/µL and the LOQ value was equal to 6.36 ng/µL. 

### 4.4. Bioassays

#### 4.4.1. Reversal Feeding Experiments

A reversal feeding experiment was designed to correlate the release of α-terpineol to the behavior of feeding males. Two sets of male weevils were prepared, males feeding on 8 g soursop pieces (initial feeding males) and males without food (no-feeding males). Each insect group contained 40 males, and each group became a replicate. Fifteen replicates were performed for initial feeding and no-feeding male weevils. The volatile collections were carried out as previously described for dynamic headspace. After the volatile collection time was reached, the insects were removed from the flask and transferred to clean ones. Then, food was not provided to the initial feeding males. On the other hand, 8 g of soursop was provided to the previous no-feeding males. The volatile captures started again under the same conditions. The samples were then analyzed, quantified by GC-MS, and compared to each other.

#### 4.4.2. Behavioral Response of Males and Female Weevils to Volatile Compounds

The behavior of males and females Annonaceae fruits weevil was evaluated through their response to different volatile stimuli in two-choice olfactometry bioassays. Choice combinations are presented in Table 1. The olfactometer was a Y-maze model: its main arm was 12 cm long with 2.5 cm of internal diameter (ID); the two choice arms were 10 cm long and 2.5 cm of ID separated at a 45° angle. The bioassay room was covered with black matte paper to avoid light reflections on the walls. Due to the biological behavior of the anonacea weevils, the olfactometer was elevated 6 cm from the table using a white styrofoam bar, just under the end of the choice arms. This elevation also caused a 4.5 cm elevation between the working bench and the arms’ union point of the olfactometer (Supplementary Figure S1). The odor sources were placed in 650 mL Drechsel gas washing bottles. The airstream flow used to carry the odor source volatiles from the release point to the corresponding election arm was set the same as for dynamic headspace. When male or female weevils were used as odor source or part of the odor source, they were added as groups of 20 insects. When α-terpineol was tested alone or combined with soursop, it was released from a 4 cm diameter filter paper (Whatman N° 2). The filter paper was previously loaded with 100 µL of a 4 ng/µL α-terpineol hexane solution. The hexane was left to dry for 30 s. The circle paper with α-terpineol was exchanged every 3 tested weevils. In all cases, 2 g of soursop were used in these experiments. Bottles containing the odor sources were also covered with black matte paper to avoid visual and light interference in the bioassays. The light was provided with a white light bar placed in the center of the olfactometry room’s ceiling. The olfactometry system was placed right under the light source. To avoid light reflections on the olfactometer glass, a 1 cm thick white styrofoam plate was placed 10 cm above the olfactometer. That is, the styrofoam plate was located between the light source and the olfactometer, thus, getting indirect illumination.

Groups of 15 insects were tested to record the odor preference of the Annonaceae fruits weevil. Each group of 15 male or female weevils was considered one replicate. There were 4 replicates for males and females tested against different odor elections (15 × 4 × 2 = 120 insects tested per treatment) (Table 1). A single insect at time was released 2 cm inside the main arm of the Y-maze olfactometer. Subsequently, the behavior and choice of the insect were observed and annotated no longer than 10 min. An odor source choice was scored when the insect walked more than half of one of the choice arms and stayed there longer than 1 min. The data was reported as the percentage of weevils selecting a specific odor source. The data represented the choice average of four replicates ± standard error.

All tested insects were subjected to a fasting period of 24 h before being used in a two-choice olfactometry assay. The arms of the olfactometer were rotated every five insects to avoid position effects on arm selection. Additionally, the olfactometer was replaced every two replicates to avoid residual odor interference. Before starting odor tests, the air in both arms was used to check there was no specific selection caused by light reflections or remnant volatiles in the system. Four groups of 20 insects (males or females) were selected and prepared to test one whole treatment or comparison. The already used insects were fed and left to recover during 48 h. The recovered insects were mixed with other non-used insects and randomly select again to form 4 new testing groups. The selection percentage values were calculated only from responding insects (15 insects). Nonetheless, the non-responding insects’ percentage was also recorded and reported. The replicates for one treatment. To test remaining hexane in the filter paper effects, clean 4 cm diameter filter paper (Whatman N° 2) were wet with 100 µL of pure hexane. The solvent was left to dry for 30 s and selection between air vs. dried filter paper was assayed. Moreover, the dried filter papers were placed in both arms to determine the presence of selection patterns by male and female weevils.

### 4.5. Statistical Analysis 

All data was subjected to normality and variance homogeneity tests. For olfactometry assays, one odor source vs. air, a one-way ANOVA was used, followed by a Tukey test (α = 0.05). A G-test analyzed olfactometry assays challenging two odor sources at once. Reversal feeding experiment data (measurements over the same specimens) was treated as pairwise samples analyzed by a U-test. For matrix recovering effects, the concentration effects of the extracts were tested by a 2 tails *t*-test (*t* < 0.05). On the other hand, a Kruskal–Wallis test was performed for data without normal distribution or homogeneous variance. The post hoc analysis was performed with Bonferroni correction (non-response olfactometry data). Statistical differences among non-responding weevils were determined with an ANOVA test. All analyzes were performed in R software [52].

## Figures and Tables

**Table 1:** Odor source combinations used in olfactometry assays. For all bioassays, when the odor source required it, 2 g of soursop were used. All male and female groups contained 20 specimens. For combinations including α-terpineol, 100 µL of a 4 ng/µL solution were added.

One Odor Source Trials	
Air	Soursop
Air	Males
Air	Females
Air	α-terpineol
Air	soursop + α-terpineol
Air	soursop + males
Air	soursop + females
**Two Odor Source Trials**	
Soursop	soursop + males
Soursop	soursop + females
Soursop	soursop + α-terpineol
soursop + females	soursop + males
Females	Males

## Data Availability

Not applicable.

## Supplementary Materials

The following are available online. Table S1: GC-MS identification of soursop volatiles trapped by dynamic headspace and solid phase microextraction. Figure S1: Olfactometry system diagram.
