# Supplementary material for: α-Terpineol: An Aggregation Pheromone in Optatus palmaris (Coleoptera: Curculionidae) (Pascoe, 1889) Enhanced by Its Host-Plant Volatiles
Source: Molecules. 2021 May 12;26(10):2861. doi: 10.3390/molecules26102861 (PMC8150320; doi:10.3390/molecules26102861)
Supplement: Supplementary file 1 [file molecules-26-02861-s001.zip › molecules-1185351-supplementary.pdf]

Supplementary table 1. GC-MS identification of soursop volatiles trapped by dynamic headspace and solid phase microextraction.

Compound	Technique	Formula	Score match (%)[a]	DB mass[b]	CAS[c]
β-elemene	SPME	C15H24	76.30	204.2	110823-68-2
cis-*β*-farnesene	AD	C15H24	77.51	204.2	28973-97-9
trans-*β*-ionone	SPME	C13H20O	77.51	192.2	79-77-6
benzophenone	AD/SPME	C13H10O	79.57	182.1	119-61-9
Octanal	AD/SPME	C8H16O	79.81	128.1	124-13-0
β-terpinene	SPME	C10H16	80.59	136.1	99-84-3
oxalic acid allyl nonyl ester	AD	C14H24O4	82.46	256.2	1000309-23-7
Decanal	AD/SPME	C10H20O	83.51	156.2	112-31-2
(*Z*)-2-Dodecenol	AD/SPME	C15H24	84.24	204.2	5208-59-3
(-)-*β*-bourbonene	AD/SPME	C15H24	84.24	204.2	5208-59-3
β-caryophyllene*	AD/SPME	C15H24	84.57	204.2	118-65-0
2,5-hexanedione	AD/SPME	C6H10O2	85.59	114.1	110-13-4
2-hexenoic acid	AD/SPME	C7H12O2	85.98	128.1	2396-77-2
D-limonene*	AD	C10H16	86.15	136.1	5989-54-8
pentanedioic acid	AD/SPME	C7H12O4	86.16	160.1	1119-40-0
nonanal*	AD/SPME	C9H18O	86.84	142.1	124-19-6

*Means that the compound was compared with a standard compound. [a] Match percentage between the mass spectra of the compound and the NIST library (V. 2014). [b] Represents the higher m/z values observed in the compound mass spectra. [c] Chemical abstracts service registry number.

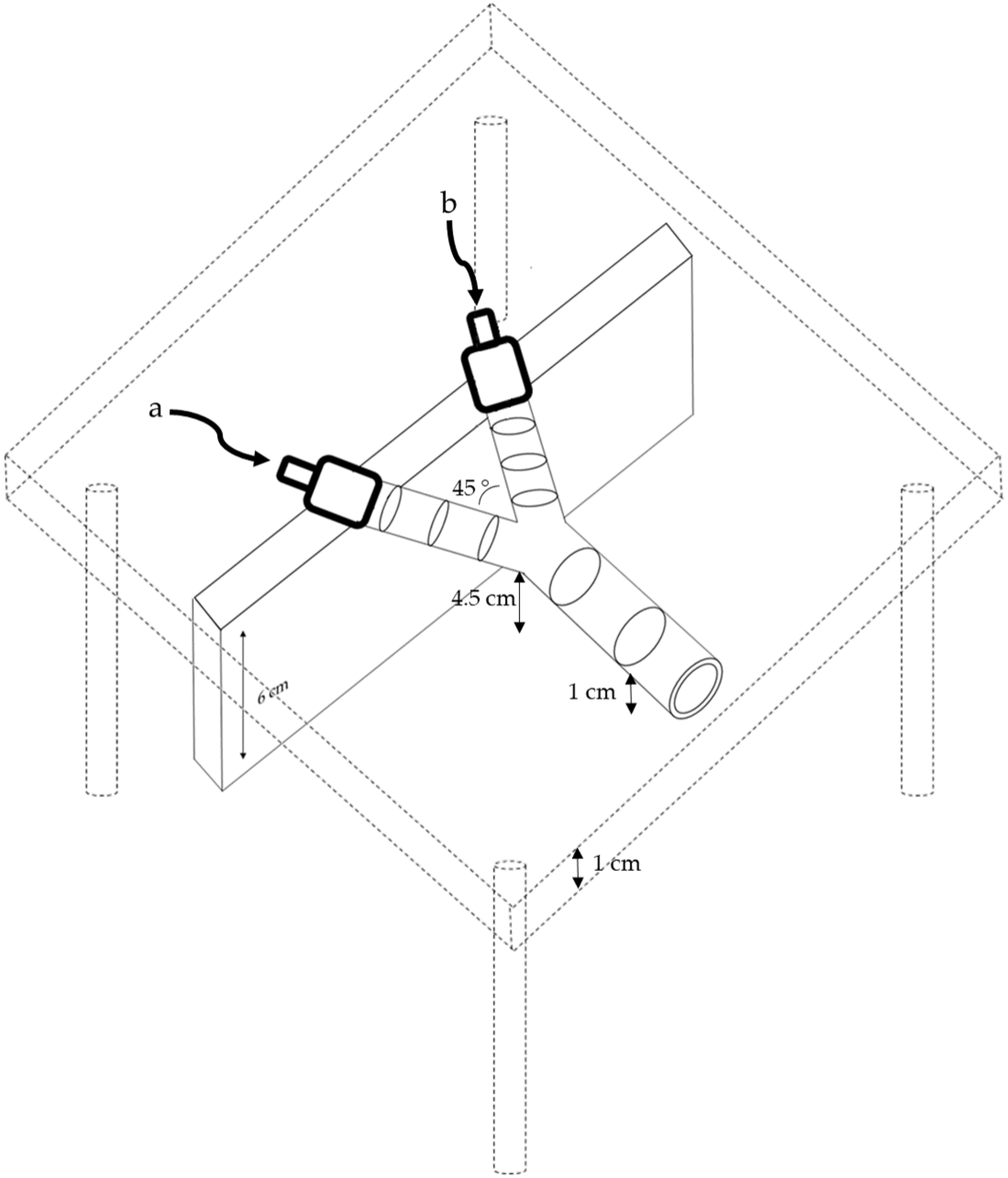

Supplementary Figure S1. Olfactometry system diagram. The olfactometer consisted of a Y-maze olfactometer with 45 ° of separation between the two flow arms. The olfactometer was placed on top of a 6 cm styrofoam bar to cause an inclination of the system. The height between the union point of the olfactometer arms and the working bench was 4.5 cm. The olfactometry system was placed under two with light bars. To avoid light reflections on the walls, the room was covered with black mate paper. To avoid light reflections on the glass olfactometer and homogenize the light dispersion over the olfactometer, a 1 cm thick styrofoam bar was placed over the system. **a** and **b** represent air flow input to the system. In one stimulus experiments, one of the arms supplies filtered air and the other one an odor stimulus. In two stimulus experiments, both arms supply odor stimulus.
